# Study on the Efficacy and Pharmacological Mechanism of Innate Immune STING Pathway Regulators in the Treatment of Ischemic Brain Injury

**DOI:** 10.3390/ph18121775

**Published:** 2025-11-21

**Authors:** Chang Liu, Xiaoqing Wang, Yueru Zhang, Songli Yu, Xiangshi Tan

**Affiliations:** 1 Department of Chemistry, Fudan University, Shanghai 200433, China; 19110220106@fudan.edu.cn (C.L.); 20110220119@fudan.edu.cn (X.W.); 22110220101@fudan.edu.cn (S.Y.); 2 Hangzhou Orenstar Biomed Co., Ltd., Hangzhou 314000, China; orenstar@163.com

**Keywords:** innate immune regulation, ischemic stroke, ST909, STING/IRF3/PI3K/AKT pathway, microglia

## Abstract

**Background/Objectives**: The efficacy of ST909, an innate immune cGAS/STING/IRF3 pathway regulator, against ischemic brain injury was investigated, and its pharmacological mechanism was elucidated. **Methods**: The efficacy and pharmacological mechanism of ST909 in ischemic brain injury were evaluated using the middle cerebral artery occlusion (MCAO) rat model, with brain tissue staining, MRI, behavioral tests (balance beam, screen), and ELISA detection of brain injury markers (neuron-specific enolase [NSE], homocysteine [Hcy], and S100β). **Results**: ST909 significantly reduces cerebral ischemic area, restores blood–brain barrier integrity, and improves neuronal function, outperforming clinical drugs (3-n-butylphthalide and edaravone) in preclinical models. ST909 markedly reduces neuroinflammation while upregulating neurotrophic factors (e.g., BDNF, NGF) in brain tissue. Through PI3K/Akt pathway activation in microglia, ST909 induces *M1*-to-*M2* phenotype polarization, rebalances the *M1*/*M2* ratio, and enhances secretion of anti-inflammatory cytokines and neurotrophic factors, thereby reducing chronic inflammation and promoting neurological recovery. These findings elucidate ST909’s potential pharmacological mechanism against ischemic brain injury, involving microglial polarization via STING/IRF3 and PI3K/Akt pathway. **Conclusions**: ST909 has a significant pharmacological effect on improving the ischemic area of the brain and repairing the function of the brain neuronal tissues. Targeting the STING/IRF3 pathway, ST909 exhibits neurorestorative potential in post-ischemic brain injury recovery.

## 1. Introduction

Stroke is a leading global cause of mortality and chronic disability. Approximately 22 million people worldwide experience stroke annually. stroke represents the primary cause of adult mortality and disability [1]. From 2010 to 2019, ischemic stroke incidence increased from 110 to 125.6 cases per 100,000 population. Accelerating population aging has driven exponential growth in stroke-related economic burdens. Ischemic stroke constitutes >80% of cases, with significantly elevated incidence, disability rates, mortality, and recurrence compared to hemorrhagic subtypes [2]. Thrombotic events underlie most ischemic strokes, carrying 10% one-year mortality and 25% severe disability rates despite treatment [3]. Current clinical interventions remain limited in efficacy, particularly beyond acute treatment windows.

Ischemic stroke progression occurs in three distinct temporal phases: acute (0–24 h post-ischemia), subacute (days 2–7), early subacute (weeks 2–8) and chronic (≥8 weeks), each characterized by specific pathophysiological events. During the acute phase, the ischemic core generates excessive reactive oxygen species (ROS) and pro-inflammatory cytokines (TNF-α, IL-1β). These mediators upregulate endothelial adhesion molecules (VCAM-1, ICAM-1), enabling leukocyte extravasation across the blood–brain barrier (BBB) via paracellular transmigration. The subacute phase features amplified neuroinflammation as infiltrated neutrophils and activated microglia secrete additional ROS, matrix metalloproteinases (MMPs), and chemokines (CXCL8, CCL2), culminating in BBB disruption and caspase-3-mediated neuronal apoptosis. The chronic phase is characterized by persistent neurological deficits, including motor dysfunction, cognitive decline, and depression.

Currently, the treatment of ischemic brain injury primarily focuses on improving cerebral blood circulation and protecting the nervous system [4]. The drugs commonly used in clinical practice include thrombolytic agents such as reteplase (r-PA) and tenecteplase (TNK-t-PA), as well as neuroprotective drugs like edaravone (EDA), 3-n-butylphthalide (NBT), and phosphatidylcholine [5,6]. Intravenous thrombolysis is the most effective pharmacological treatment for acute ischemic stroke; however, due to the narrow therapeutic time window and other limitations, only a small proportion of eligible patients receive thrombolysis. Recent studies indicate that approximately 20% of patients present to the emergency department within 3 h of symptom onset, 12.6% meet the eligibility criteria for thrombolysis, yet only 2.4% ultimately receive thrombolytic therapy [7,8,9]. Additionally, the rate of thrombolytic administration is critical. Excessive infusion rates may induce secondary damage to the infarcted brain tissue, potentially leading to vascular rupture and intracranial hemorrhage in severe cases [8]. These limitations restrict the widespread clinical application of thrombolytic agents [10]. Neuroprotective agents like EDA and NBT are typically administered within 48 h post-symptom onset, with a treatment duration not exceeding 14 days. Their primary mechanism involves scavenging free radicals and mitigating neuroinflammation triggered by cerebral ischemia [11]. However, their efficacy in promoting functional recovery of damaged brain tissue remains limited, highlighting an unmet clinical need for therapies that enhance post-ischemic neural repair.

Neuroinflammation, a key contributor to ischemic brain injury, exacerbates tissue damage by disrupting the blood–brain barrier (BBB), inducing oxidative stress, and triggering widespread neuronal apoptosis through microglial overactivation. Conversely, microglial activation also plays an essential role in tissue repair and neuroplasticity [12]. Thus, maintaining the equilibrium between pro-inflammatory (*M1*) and anti-inflammatory (*M2*) microglial polarization states is critical. Targeted modulation of microglial activity to enhance their reparative functions—such as anti-inflammatory factor secretion, neurotrophic factor release, and polarization balance—holds therapeutic potential for ischemic brain injury recovery. The STING/IRF3 pathway has emerged as a key regulator of post-ischemic microglial activation. While extensively studied in ischemic stroke, the STING/IRF3 pathway’s role remains controversial, with evidence supporting both detrimental and beneficial effects. Some studies demonstrate that cGAS/STING/IRF3 pathway activation aggravates brain injury. Genetic knocking out or pharmacological inhibition of this pathway attenuates pro-inflammatory microglial polarization, reduces neuronal apoptosis, and improves functional outcomes in preclinical models [13,14]. Mechanistically, post-stroke cytosolic DNA accumulation activates cGAS, triggering the STING-dependent production of type I interferons (IFNs) and pro-inflammatory cytokines that drive microglial polarization toward a neurotoxic (*M1*) phenotype [15]. Conversely, the cGAS/STING/IRF3 pathway also mediates reparative processes post-ischemia. STING-induced interferon-β (IFN-β) stimulates angiogenesis and neurogenesis, facilitating neural repair and functional recovery in the ischemic penumbra [16].

These divergent findings highlight the cGAS/STING/IRF3 pathway’s pivotal role in post-stroke immune regulation, orchestrating immune cell activation, cytokine dynamics, neuroinflammation, apoptosis, and tissue repair during stroke recovery. Thus, targeted modulation of STING/IRF3 pathway may promote neural repair in stroke recovery. This study employs a rat middle cerebral artery occlusion (MCAO) model to examine ST909—a novel STING pathway modulator—and its therapeutic mechanisms against ischemic stroke. Cyclic dinucleotides (CDNs) are a class of STING agonists. ST909, a CDN, is an endogenous second messenger that directly activates the STING signaling pathway. It functions as a critical alarm signal in mammals: upon cellular infection or damage, it triggers the host immune response. ST909 was enzymatically synthesized using an in vitro bioreactor system. ST909 was enzymatically synthesized using an in vitro bioreactor system. Our findings will inform the development of therapeutics that restore neuroimmune homeostasis, mitigate neuroinflammation, and enhance functional recovery after stroke.

## 2. Results

### 2.1. Exploration of Dose–Response Relationship of ST909 in MCAO Rats with Ischemic Brain Injury

**Figure 1:**
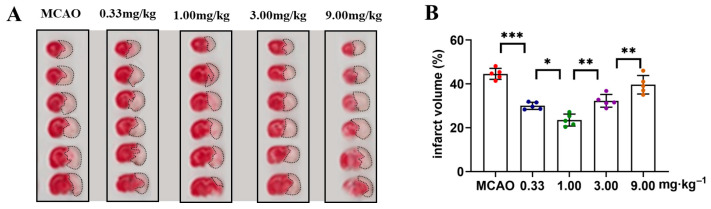
TTC staining analysis of cerebral infarction volume in ST909-treated MCAO rats across dose gradients: (**A**) Representative TTC-stained coronal brain sections from different treatment groups (doses: 0.33, 1.00, 3.00, 9.00 mg/kg). The unlabeled version is shown in Figure S1. White areas indicate infarcted tissue. (**B**) Quantitative analysis of infarct volume (One-way ANOVA test in (**B**), *n* = 5, the results as shown as mean ± S.D. ***, *p* < 0.001. **, *p* < 0.01. *, *p* < 0.05).

The dose–response relationship of ST909 for ischemic brain injury of MCAO rates was firstly investigated in this study. Four dose levels (0.33, 1.00, 3.00, and 9.00 mg/kg) were tested. Following treatment, brain tissues were sectioned and stained to assess ischemic damage area (Figure 1A). The results showed that the efficacy of ST909 in ischemic brain injury exhibited a significant dose effect dependence relationship, and conflicting efficacy results were observed at low and high doses. As shown in Figure 1, ST909 at low doses (0.33–1.00 mg/kg) promoted recovery of ischemic damage area, showing a dose-dependent reduction in infarct volume. Therapeutic efficacy presented a positive correlation with concentration in this low dose range. In contrast, high doses (3.00–9.00 mg/kg) of ST909 produced poor effects. When the dose exceeds 1.00 mg/kg, the infarct volume gradually increases with dose, indicating an opposite dose–response relationship (Figure 1B). These findings indicate that the pharmacological mechanism of ST909 involves complex nonlinear dose–response characteristics. Based on these dose–response results, we optimized the dose of 1.00 mg/kg ST909 for its efficacy and pharmacological mechanism in this study.

### 2.2. Effect of ST909 on Recovery from Ischemic Brain Injury in MCAO Model Rats

**Figure 2:**
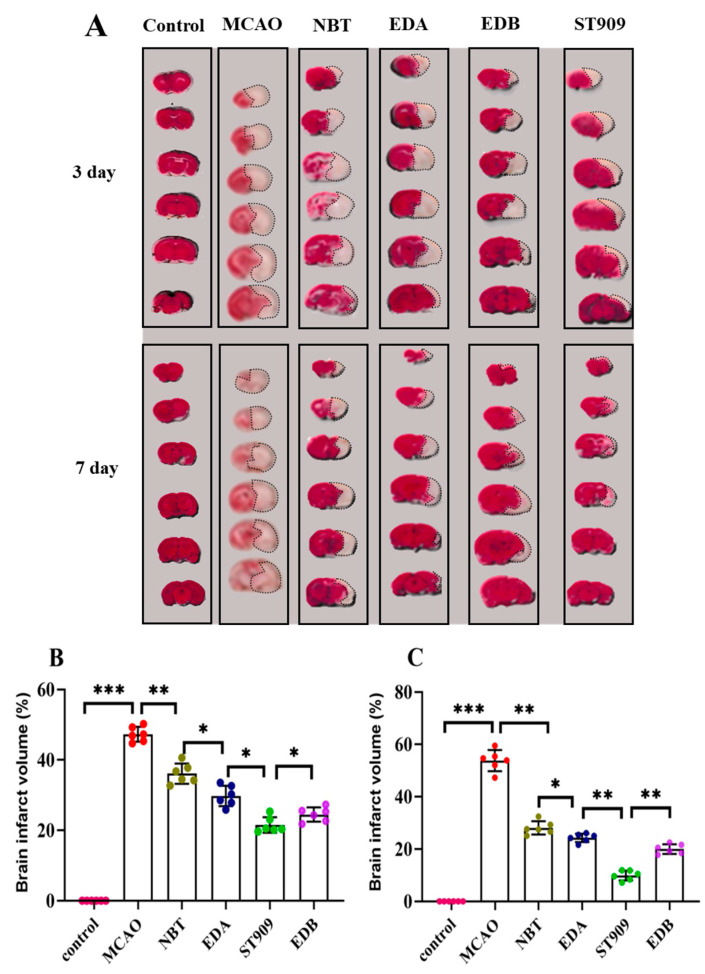
TTC staining analysis of cerebral infarction volume in different treatment groups: (**A**) Representative TTC-stained coronal brain sections from different treatment groups. White areas indicate infarcted tissue. The unlabeled version is shown in Figure S2. (**B**) Quantitative analysis of infarct volume in 3 days; (**C**) Quantitative analysis of infarct volume in 7 days. (One-way ANOVA test in (**B**,**C**), *n* = 6; the results as shown as mean ± S.D. ***, *p* < 0.001. **, *p* < 0.01. *, *p* < 0.05). Data for the 21-day drug treatment are provided in Figure S2 and Table S1.As presented in Figure 2B, we chose first-line clinical drugs NBT and EDA as positive controls. Administration of 20 mg/kg/d NBT via tail vein injection significantly reduced the ischemic area to 35.2 ± 5.7% after 3 days. Similarly, 6 mg/kg/d EDA treatment for 3 days decreased the ischemic area to 27.2 ± 4.3%, showing a statistically significant difference compared to the MCAO group (*p* < 0.01). In the ST909 group, 3-day administration further reduced the ischemic area to 21.7 ± 5.1%, indicating that ST909′s neuroprotective effect in the subacute phase was significantly superior to both positive controls (*p* < 0.05).

In order to investigate the pharmacological effects of ST909 on ischemic brain injury, we selected dosing cycles of 3 and 7 days. After the dosing cycle was completed, the rats were euthanized, and brain slices were collected for TTC staining. The results are shown in Figure 2. The MCAO model group (G1) exhibited a large cerebral infarct area, with an ischemic region of 47.3 ± 1.9% in 3 days, confirming the success of cerebral ischemia–reperfusion surgery.

Based on these findings, we further evaluated ST909′s efficacy in the late acute phase (7 days post-ischemia). As shown in Figure 2C, after 7 days of treatment, the infract areas in the NBT and EDA groups decreased to 29.5 ± 7.2% and 23.3 ± 5.7%, respectively, demonstrating improvement over the subacute phase. Notably, ST909 treatment for 7 days reduced the infract area to 13.1 ± 4.6%, representing only 44.8% and 56.2% of the infarct sizes observed with NBT and EDA, respectively. ST909′s therapeutic effect was significantly better than both positive drugs (*p* < 0.01). We further evaluated ST909 compared to clinical combination therapy (edaravone + (+)-borneol, EDB). Both regimens were administered intravenously for 3 consecutive days to assess subacute-phase efficacy (Figure 2B,C). The EDB group exhibited 26.9 ± 7.4% ischemic area, whereas ST909 showed significantly reduced infarction (21.7 ± 5.1%, *p* < 0.05). This superiority persisted in the late acute phase (7 days), with ischemic areas of 19.2 ± 6.8% versus 13.1 ± 4.6%, respectively. These findings demonstrate that the ST909 significantly outperformed EDB at all stages, establishing its superior neuroprotective efficacy.

ST909 outperforms NBT, EDA and EDB in reducing ischemic brain injury across all recovery phases, with significant improvements in infarct volume and motor function.

### 2.3. Therapeutic Effect of ST909 by MRI Brain Imaging

At 24 h, 3 days, and 7 days after cerebral ischemia induction in MCAO rats, we performed coronal-plane magnetic resonance T2WI and DWI imaging sequences on MCAO rats in different treatment groups. T2WI was used to assess changes in brain edema and ischemic regions, while DWI was used to calculate the relative apparent diffusion coefficient (rADC), specifically NFR and PNR. NFR reflects the recovery of the lesion area relative to the normal tissue, whereas PNR reflects the recovery of the penumbra relative to the normal tissue.

**Figure 3:**
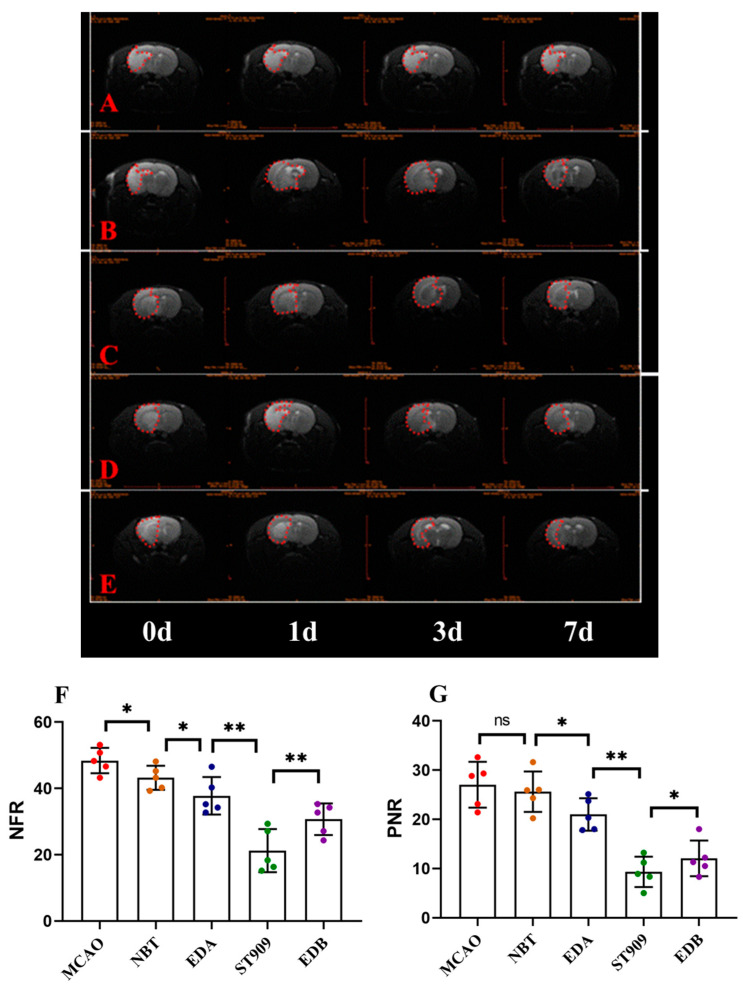
Representative Brain MRI images (**A**) MCAO; (**B**) NBT; (**C**) EDA; (**D**) EDB; (**E**) ST909 and relative apparent diffusion coefficient (rADC) value comparison (7 days) (**F**) NFR; (**G**) PNR. The unlabeled version is shown in Figure S3. One-way ANOVA test in (**F**,**G**), *n* = 5; the results as shown as mean ± S.D. **, *p* < 0.01; *, *p* < 0.05; ns, *p* > 0.05). The red dotted area represents the brain edema area. The normalized functional recovery (NFR) index reflects the recovery of the lesion area relative to the normal area, while the penumbra-to-normal ratio (PNR) reflects the recovery of the penumbra relative to the normal area. The apparent hemispheric asymmetry observed in MRI images results from pathological cerebral edema secondary to ischemic injury.

The T2WI results are shown in Figure 3, where the hyperintense area represents cerebral infarction, and the asymmetry indicates the degree of cerebral edema. In the model group (Figure 3A), at 24 h post-ischemia, the hyperintense region (infarct zone) progressively expanded and partially involved the cortex. Compared with the contralateral (right) hemisphere, the left hemisphere showed marked deformation, indicating severe cerebral edema. The EDA-treated group exhibited a significant reduction in ischemic area and cerebral edema compared to the model group. No significant difference in ischemic area was observed between the ST909, NBT, EDB and EDA groups at 24 h (*p* > 0.05), with ST909 showing a marginally larger infarct size. However, with prolonged treatment, the ST909 group demonstrated a significant reduction in ischemic area and resolution of cerebral edema compared to the NBT, EDB and EDA groups.

These findings were further supported by rADC values derived from DWI (Table 1, Figure 3F,G). At 24 h post-ischemia, the MCAO group showed NFR and PNR values of 47.3 ± 5.1 and 24.4 ± 6.3, respectively. The EDA group exhibited lower NFR (40.9 ± 4.7) and PNR (23.6 ± 1.4) than the model group (*p* < 0.05), suggesting mild improvement in ischemic recovery. After 24 h treatment, the ST909 group had comparable NFR (40.1 ± 3.2) and PNR (22.9 ± 2.8) with the EDA group (*p* > 0.05). By day 7, however, the ST909 group showed significantly lower NFR (25.5 ± 3.7) and PNR (8.96 ± 2.5) than the EDA group (*p* < 0.01), along with a pronounced decrease in rADC values. Compared with the EDA groups, ST909 also showed similar trends of change.

Similarly, MRI scans were performed on rats receiving combination therapies: EDB and ST909 (Figure 3D,F). T2WI results demonstrated that combination therapies effectively suppressed cerebral edema within 24 h, with significantly smaller hyperintense regions compared to monotherapy groups. By day 7, bilateral hemispheric symmetry was restored in both groups, with no observable edema-induced deformation. Compared to EDB, the ST909 group exhibited complete resolution of hyperintense signals, demonstrating superior therapeutic efficacy (*p* < 0.01).

These findings were validated using DWI-derived rADC values (Table 1, Figure 3F,G). At day 7, the EDB group showed NFR (29.5 ± 4.7) and PNR (12.9 ± 1.6), representing significant improvement versus the model group (*p* < 0.01). From day 3 onward, rADC values decreased markedly, showing significant differences versus EDB (*p* < 0.05), demonstrating superior long-term efficacy compared to this clinical standard.

The therapeutic time window was characterized through serial MRI monitoring of cerebral ischemia recovery. First-line clinical regimens (EDA alone or EDB) significantly reduced infarct volume and cerebral edema, though treatment effects plateaued over time. However, ST909-containing regimens showed limited early efficacy (0–24 h, *p* > 0.05), with significant improvement emerging by 72 h (*p* < 0.01). These findings suggest distinct mechanisms of action between ST909 and conventional neuroprotectants like EDA.

### 2.4. ST909 Reduces Ischemic Brain Injury by Stroke Biomarkers

Next, we analyzed stroke biomarkers in peripheral blood. Neuron-specific enolase (NSE), S-100β, and homocysteine (Hcy) are localized in distinct neural tissue regions. Their circulating levels showed positive correlation with ischemic injury severity following cerebral infarction. Elevated peripheral NSE levels specifically reflected neuronal glycolysis dysregulation and tissue damage extent. Pathological processes including cerebral edema, neuronal integrity impairment, and metabolic dysfunction facilitate NSE translocation from cerebrospinal fluid into systemic circulation through the compromised blood–brain barrier [17].

The central nervous system-specific protein S-100β, a low molecular weight calcium-binding protein, serves as a sensitive neuroglial damage marker [17]. Homocysteine (Hcy), an established vascular risk factor, exerts endothelial toxicity through multiple mechanisms: disrupting lipoprotein oxidation pathways, promoting platelet aggregation, and inducing thrombogenesis [18].

**Figure 4:**
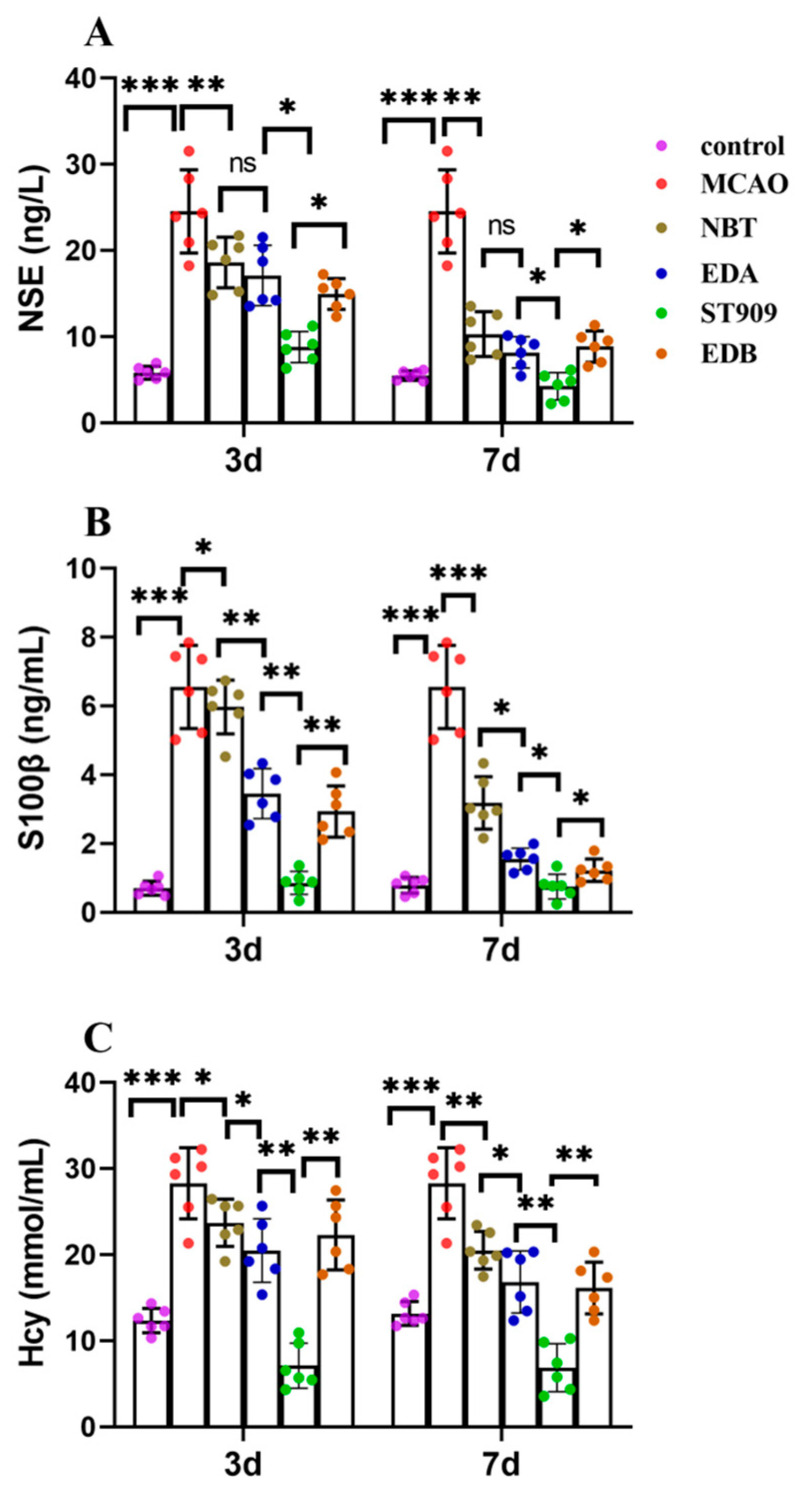
Comparison of serum biomarkers (NSE, S100β, Hcy) between 3 and 7 days after ischemic stroke in different treatment group. The statistics were conducted with the One-way ANOVA test (**A**) NSE; (**B**) S100β; (**C**) Hcy. *n* = 6; the results as shown as mean ± S.D. ***, *p* < 0.001; **, *p* < 0.01; *, *p* < 0.05; ns, *p* > 0.05).

In MCAO-model rats, three days post-ischemia revealed marked biomarker elevation: NSE at 26.3 ± 2.1 ng/L (vs. normal 6.9 ± 1.4 ng/L), S-100β at 6.18 ± 1.17 ng/mL (vs. normal 0.53 ± 0.21 ng/mL) [19], and Hcy at 28.3 ± 2.8 μmol/L (vs. normal 15.3 ± 4.9 μmol/L) [20]. Seven-day treatment with NBT, EDB or EDA restored biomarkers close to normal levels, suggesting neuronal repair and cerebral microenvironment homeostasis restoration. Remarkably, ST909 administration achieved rapid biomarker normalization within three days (NSE: 9.35 ± 0.85 ng/L; S-100β: 1.18 ± 0.29 ng/mL; Hcy: 12.3 ± 0.6 μmol/L), reaching normal levels by day 7 (Figure 4A–C).

ELISA-based quantification revealed that clinical first-line drugs required seven days to restore serum biomarkers to safe thresholds. In contrast, ST909 achieved comparable safety thresholds within three days. By day seven, biomarker levels in treatment groups showed no statistically significant difference from healthy controls. These findings demonstrate that ST909 exhibited superior therapeutic efficacy to clinical first-line drugs.

### 2.5. ST909 Significantly Improved Motor Function in MCAO Rats with Ischemic Brain Injury

**Figure 5:**
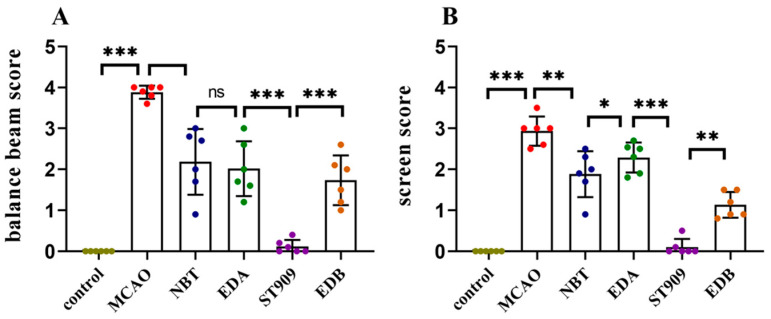
Statistical comparison of behavioral scores between treatment groups. The statistics were conducted with the Two-way ANOVA test (**A**) balance beam; (**B**) screen. *n* = 6; the results as shown as mean ± S.D. ***, *p* < 0.001.**, *p* < 0.01. *, *p* < 0.05; ns, *p* > 0.05).

In the balance beam test, we assessed the post-surgical balance ability of rats (Figure 5A). A lower score indicates worse motor function. MCAO group rats had severely impaired balance, failing to traverse the beam. EDA and EDB group rats could cross the beam but showed >50% fall rates. The NBT group performed better (fall rate < 50%) than the previous two groups. ST909-treated rats successfully traversed the beam without falling.

The screen test assessed post-surgical forelimb grip strength (Figure 5B). MCAO group rats dropped immediately upon grid rotation. EDA group maintained grip for 5 s before falling. NBT and EDB group rats slid but did not drop. ST909 group maintained grip for >5 s.

These results demonstrate that ST909 are superior to first-line clinical drugs (e.g., EDA) in restoring motor function after ischemic injury.

### 2.6. ST909 Attenuates Neuroinflammation in MCAO Rats with Ischemic Brain Injury

The pathogenesis of ischemic brain injury involves chronic neuroinflammation. To elucidate ST909′s mechanism, we analyzed key inflammatory mediators. Pro-inflammatory factors TNF-α and IL-1β were significantly elevated in the MCAO control group. TNF-α, primarily secreted by activated macrophages, NK cells, and T lymphocytes [21], induces endothelial dysfunction and microvascular thrombosis. IL-1β promotes neuronal apoptosis and is upregulated in ischemic brain tissue [21].

Conversely, the expression of anti-inflammatory factors, primarily IL-4 and IL-10, was inhibited. Interleukin-4 (IL-4) has therapeutic effects on various injuries and infectious diseases. The expression of IL-4 promotes recovery from ischemic brain injury [22]. Interleukin-10 (IL-10), as an anti-inflammatory factor, can inhibit the expression of pro-inflammatory factors, alleviate the damage caused by excitatory neurotoxic substances to nerve cells, and exerts neuroprotective effects [23].

**Figure 6:**
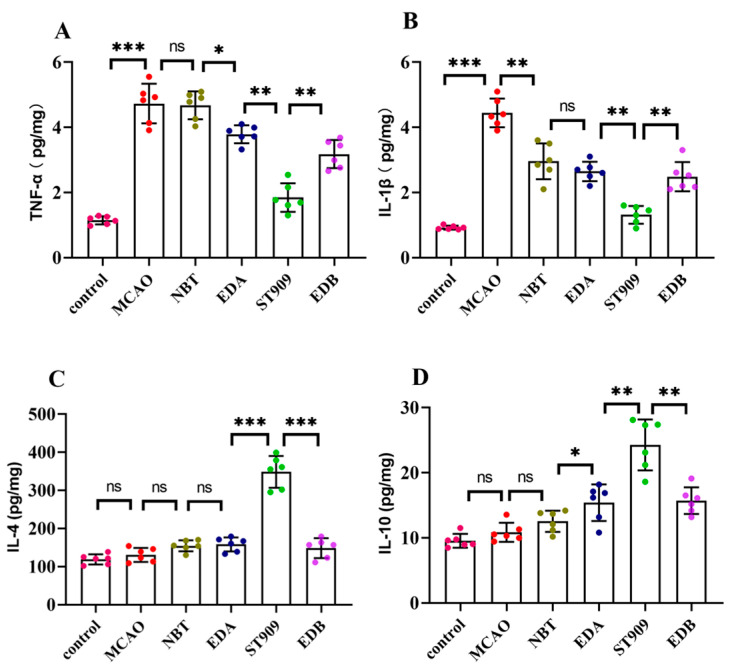
Quantification of inflammatory factors in brain tissue by ELISA. The statistics were conducted with the One-way ANOVA test (**A**) TNF-α; (**B**) IL-1β; (**C**) IL-4; (**D**) IL-10. *n* = 6; the results as shown as mean ± S.D. ***, *p* < 0.001.**, *p* < 0.01. *, *p* < 0.05; ns, *p* > 0.05).

Notably, as shown in Figure 6, conventional therapies (NBT/EDA) demonstrated limited efficacy, reducing TNF-α/IL-1β by merely 10–20% versus MCAO (*p* < 0.05). In contrast, ST909 markedly suppressed these pro-inflammatory cytokines (TNF-α/IL-1β) by 50–60% (*p* < 0.001). Regarding anti-inflammatory factors, neither IL-4 nor IL-10 showed significant changes with standard treatments (*p* > 0.05). However, ST909 regimens upregulated both IL-4 and IL-10 by >2-fold (*p* < 0.001).

The above results indicate that the ST909 containing group can significantly reduce the expression level of pro-inflammatory factors in the brain environment and upregulate the expression of anti-inflammatory factors. Therefore, we can speculate that the therapeutic effect of ST909 needle on ischemic brain injury is contributed partly by inflammation elimination.

### 2.7. ST909 Modulates Microglial Polarization from M1 to M2 Phenotype in MCAO Rats with Ischemic Brain Injury

Microglia, the resident macrophages and predominant immune cells in the central nervous system (CNS), exhibit remarkable sensitivity to alterations in the neuronal microenvironment [24]. These cells dynamically transition between the pro-inflammatory *M1* and anti-inflammatory *M2* phenotypes, thereby secreting distinct cytokines that modulate adjacent astrocytes and neurons. Following cerebral ischemia, microglia undergo phenotypic polarization from resting state to pro-inflammatory *M1* phenotype, releasing pro-inflammatory mediators, whereas the anti-inflammatory *M2* phenotype secretes anti-inflammatory cytokines.

**Figure 7:**
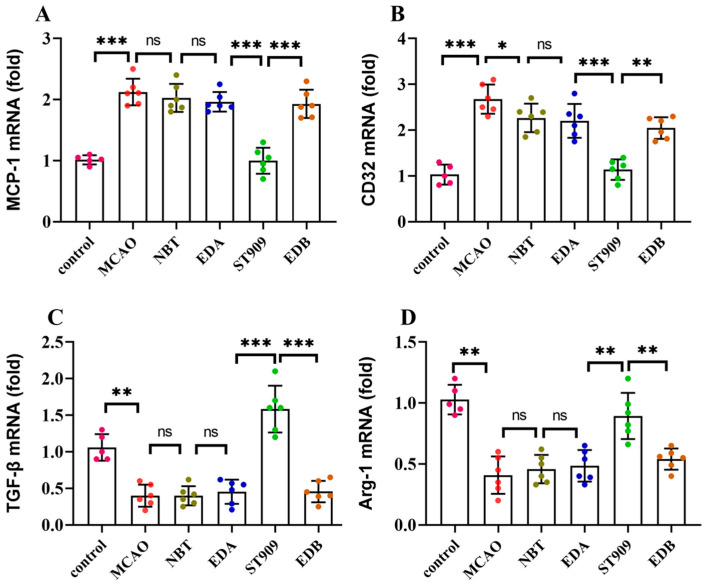
mRNA expression levels of *M1* markers (CD32, MCP-1) and *M2* markers (TGF-β, Arg-1) in microglia. The statistics were conducted with the One-way ANOVA test (**A**) MCP-1; (**B**) CD32; (**C**) TGF-β; (**D**) Arg-1. *n* = 6; the results as shown as mean ± S.D. ***, *p* < 0.001.**, *p* < 0.01. *, *p* < 0.05; ns, *p* > 0.05).

To elucidate the pharmacological mechanisms underlying ST909 modulation of neuroinflammation, we quantified the *M1*-associated markers (CD32, MCP-1) and *M2*-associated markers (TGF-β, Arg-1) using RT-qPCR. As shown in Figure 7, the MCAO group exhibited significant upregulation of *M1* markers (MCP-1 and CD32) concurrent with suppression of *M2* markers (TGF-β and Arg-1) compared with controls (*p* < 0.01). Conventional therapeutics (NBT, EDA, and EDB) demonstrated limited efficacy in modulating microglial polarization. In contrast, both ST909 therapy significantly downregulated *M1* markers (*p* < 0.001) while upregulating *M2* markers (*p* < 0.01) relative to MCAO controls.

These findings demonstrate that ST909 promotes microglial polarization from the *M1* to *M2* phenotype, thereby attenuating neuroinflammation and mitigating ischemic brain injury progression.

### 2.8. ST909 Upregulates Neurotrophic Factor Expression in MCAO Rats with Ischemic Brain Injury

The above results indicate that ST909 can regulate the transformation of microglia into the *M2* phenotype. Microglia also release a series of neurotrophic factors during the recovery phase of neuronal injury to repair damaged neurons and neuronal networks [25,26]. Brain-derived neurotrophic factor (BDNF) is the most abundant neurotrophic factor in brain tissue, playing a crucial role in the repair and functional remodeling of damaged neurons. Glial cell-derived neurotrophic factor (GDNF) exerts protective, repair, and differentiation-promoting effects on mature neurons. Nerve growth factor-1 (NGF-1) promotes neuronal repair and delays neuronal death. The neuroprotective effect of ST909 in ischemic brain injury is closely associated with the release of neurotrophic factors by microglia.

**Figure 8:**
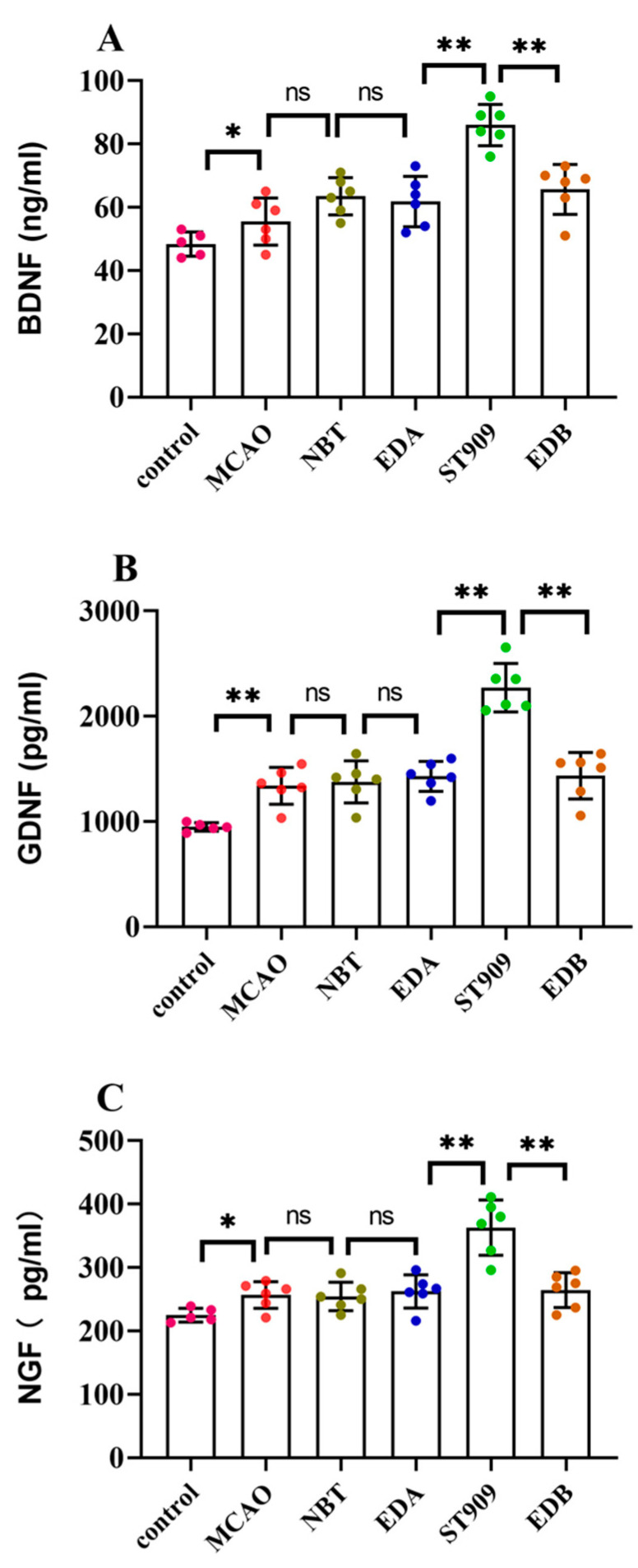
Neurotrophic factor levels in the serum of different treatment groups. The statistics were conducted with the One-way ANOVA test (**A**) BDNF; (**B**) GDNF; (**C**) NGF. *n* = 6; the results as shown as mean ± S.D. **, *p* < 0.01. *, *p* < 0.05; ns, *p* > 0.05).

As shown in Figure 8, ELISA analysis revealed that the model group exhibited only a 10–20% increase in neurotrophic factors compared to the control group. No significant difference (*p* > 0.05) was observed in neurotrophic factor expression between the model group and first-line clinical drugs, including NBT, EDA, and EDB. Compared to the model group, the ST909 groups exhibited significant increases in BDNF (34.2%), GDNF (121.2%), and NGF-1 (66.2%) concentrations (*p* < 0.01). These results demonstrate that ST909 promotes microglia polarization from the *M1* to the *M2* phenotype, upregulates neurotrophic factor expression, and enhances the repair of damaged neurons.

### 2.9. ST909-Mediated Neural Repair via STING/IRF3 and PI3K/AKT Pathway

**Figure 9:**
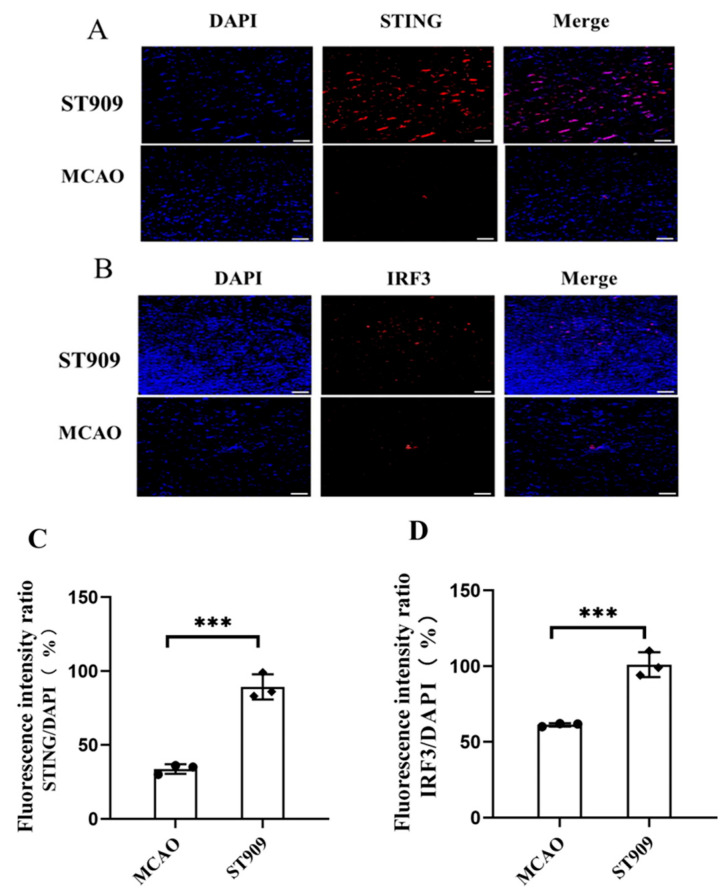
ST909 induces activation of the STING/IRF3 pathway, as demonstrated by immunofluorescence (**A**,**B**) Scale bar = 100 μm. The statistics were conducted with the ANOVA test. The semi-quantitative analysis of mean fluorescence intensity was presented as mean ± SEM (**C**,**D**) *n* = 3, ***, *p* < 0.001). The mean fluorescence intensity of STING in ST909 group is 6611, The mean fluorescence intensity of IRF3 in ST909 group is 4381.

To investigate the pharmacological mechanism of ST909, we performed immunofluorescence staining on brain tissue sections, as shown in Figure 9. STING and IRF3 expressions were markedly upregulated in the ST909-treated group than in the MCAO group. Semi-quantitative analysis revealed that ST909 treatment increased STING and IRF3 fluorescence intensity by 45% and 62%, respectively, compared with the MCAO group (*p* < 0.001).

**Figure 10:**
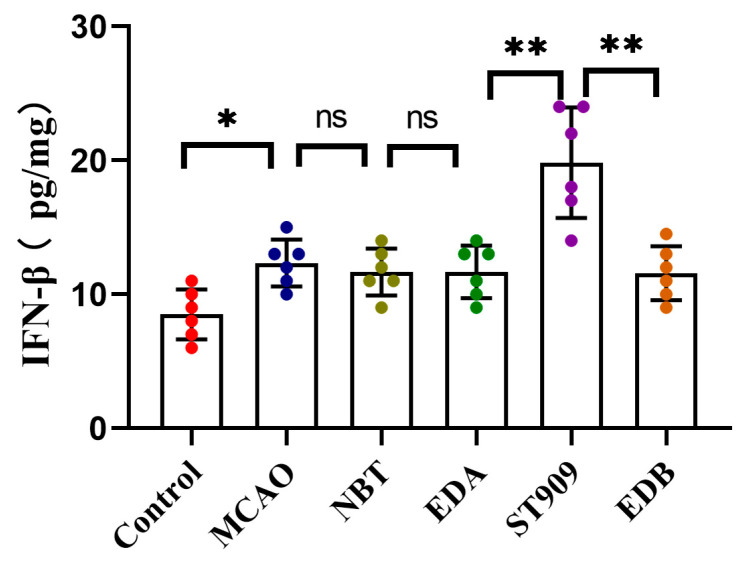
IFN-β expression in brain tissue of different treatment groups. The statistics were conducted with the One-way ANOVA test (*n* = 6, **, *p* < 0.01; *, *p* < 0.05; ns, *p* > 0.05).

At the administered dose (1 mg/kg) in this study, ST909 significantly activated the STING-IRF3 pathway during cerebral ischemia, which releases IFN-β, promoting angiogenesis and oxidative stress resistance. On the other hand, regulating the balance of *M1*/*M2* ratios and promoting microglial polarization toward the anti-inflammatory *M2* phenotype. *M2* microglia upregulated anti-inflammatory factors (IL-4, IL-10) while downregulating pro-inflammatory cytokines (TNF-α, IL-1β). Notably, TNF-α is a known activator of NF-κB in the neuronal environment [27]. Furthermore, ST909 significantly upregulated IFN-β expression (Figure 10), promoting angiogenesis and oxidative stress resistance. These results confirm that ST909 activated distinct anti-inflammatory mechanisms rather than the NF-κB pathway. Based on the dose–effect relationship results (Section 2.1), ST909 promoted recovery of ischemic areas at low concentrations but inhibited or even aggravated ischemia at high concentrations. These findings suggest that ST909 at low concentrations primarily promotes nerve repair via the STING/IRF3/IFN-β pathway. At high concentrations, the STING/IRF3/NF-κB pathway is activated, exacerbating neuroinflammation and brain injury. These results highlight the critical importance of maintaining optimal drug concentrations during treatment.

Previous studies by Tarassishin et al. demonstrated that cerebral hypoxia suppresses PI3K/Akt signaling. Phosphorylated IRF3 can induce anti-inflammatory factors (IL-10, IFN-β) via PI3K/Akt activation while persistently inhibiting pro-inflammatory cytokines (IL-1β, TNFα, IL-6) [28]. This transformation of microglia from pro-inflammatory to anti-inflammatory phenotypes was enhanced by IRF3 transcription, which plays crucial roles in neuroprotection [28]. Supporting this, Li et al. reported cGAMP-mediated upregulation of p-PI3K/p-AKT and promoting angiogenesis and neuroprotection during cerebral ischemia recovery [29].

**Figure 11:**
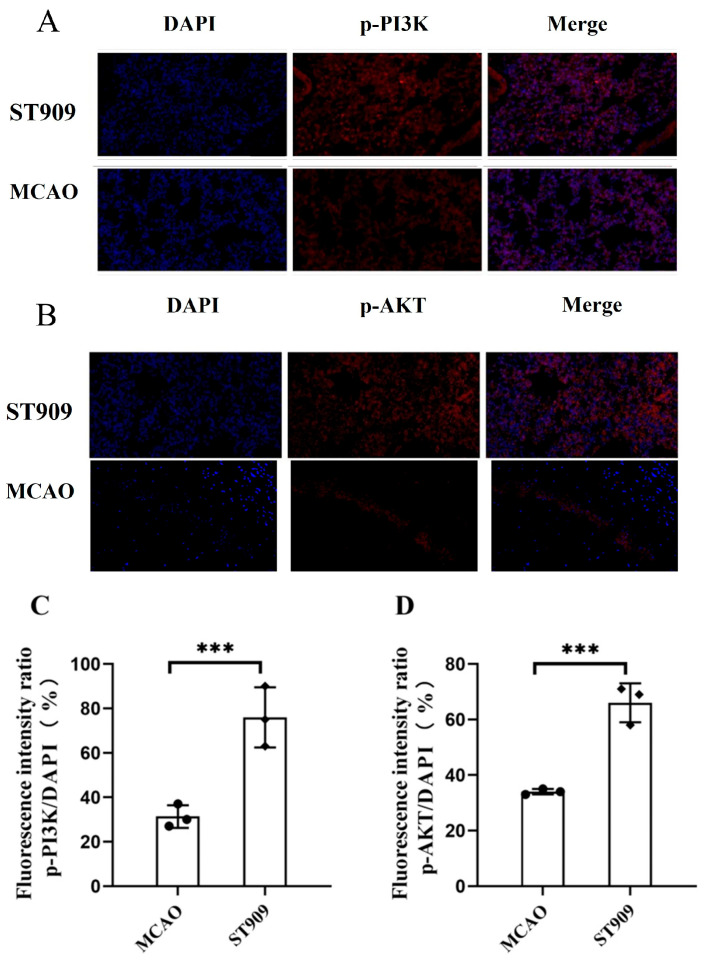
ST909 induces activation of the PI3K/AKT pathway, as demonstrated by immunofluorescence (**A**,**B**). The semi-quantitative analysis of mean fluorescence intensity was presented as mean ± SEM. Scale bar = 100 μm. The statistics were conducted with the ANOVA test (**C**,**D**) *n* = 3, ***, *p* < 0.001). The mean fluorescence intensity of P13K in ST909 group is 9356, The mean fluorescence intensity of Akt in ST909 group is 7748.

Our findings align with these reports: the phosphorylation levels of PI3K and Akt were significantly lower in the MCAO group compared to controls. In Figure 11, Semi-quantitative analysis of fluorescence intensity revealed that the p-PI3K/PI3K and p-Akt/Akt ratios were 50% and 65% higher, respectively, in the ST909 group (*p* < 0.001), indicating that ST909 acts as a secondary messenger activating both STING-IRF3 and PI3K/AKT pathways.

During the stroke recovery period, the PI3K/Akt pathway is inhibited, impairing nervous system self-repair, promoting apoptosis of damaged neurons, and inducing neuroinflammation, which exacerbates disease progression and delays neural functional recovery [30]. Meanwhile, ST909 upregulates *p*-IRF3, which directly activates the PI3K/Akt pathway—otherwise suppressed in hypoxic conditions. Upon activation by ST909, the PI3K/Akt pathway modulates microglial *M1*/*M2* polarization, suppresses inflammatory factor release, and mitigates neuroinflammation in ischemic brain tissue [31].

Additionally, activated PI3K/Akt enhances GSK-3β phosphorylation, attenuating its oxidative cascade. Concurrently, Akt activates nuclear factor E2-related factor 2 (Nrf2), facilitating antioxidant response element (ARE) binding and upregulating antioxidant protein expression. These mechanisms collectively reduce oxidative stress, thereby protecting injured brain tissue [32]. Furthermore, ST909 upregulates cytokines (e.g., IL-1α, IL-10, IFN-β) and neurotrophic factors (e.g., BDNF), enhancing axonal regeneration, neuronal differentiation, and functional recovery of damaged neurons [33].

These findings demonstrate that ST909 activates the STING/IRF3 pathway, stimulating IFN-β (Figure 10) and cytokine production to promote angiogenesis and neuroprotection. Moreover, ST909 may indirectly enhance PI3K activation during cerebral ischemia, inducing Akt phosphorylation, shifting microglia toward an anti-inflammatory phenotype, releasing anti-inflammatory mediators, upregulating neurotrophic factors, and exerting anti-apoptotic effects [34,35].

## 3. Discussion

### Pharmacological Mechanism of ST909 in the Treatment of Ischemic Brain Injury

Ischemic brain injury poses a significant clinical burden due to its high mortality and disability rates, coupled with poor prognosis. In the early stages of ischemic stroke, an inflammatory cascade occurs, exacerbating brain damage. Ischemic tissue releases abundant DAMPs, triggering immune responses that activate microglia and astrocytes. NF-κB induces massive pro-inflammatory cytokine release, activating apoptotic pathways and damaging vascular endothelial tissues within minutes to hours post-ischemia [36]. During recovery, impaired neurons compromise neurological function, with hypoxia progressively inducing neuronal apoptosis. Current evidence identifies microglia as primary mediators of neuroinflammation and apoptosis in ischemic injury [37]. However, microglia exhibit dual roles in immunity through their *M1*/*M2* polarization states. The *M2* phenotype promotes anti-inflammatory factor expression and neural repair [38], making microglia-targeted immunomodulation a promising therapeutic strategy.

**Figure 12:**
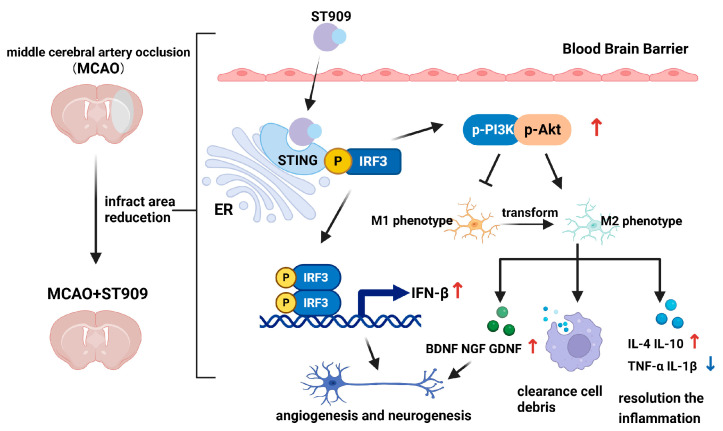
Therapeutic mechanism of ST909 in the rat model of ischemic brain injury. During the recovery phase of ischemic brain injury, ST909 activates the STING/IRF3 pathway. On the one hand, it releases IFN-β, promoting angiogenesis and oxidative stress resistance. On the other hand, phosphorylated IRF3 activates the PI3K/Akt pathway under hypoxia, regulating the balance of *M1*/*M2* ratios and promoting microglial polarization toward the anti-inflammatory *M2* phenotype. *M2* microglia secrete anti-inflammatory factors (e.g., IL-4 and IL-10), suppressing inflammatory responses and protecting healthy neural tissue. Secondly, *M2*-polarized microglia are activated and rapidly migrate to the infarct core and penumbra, engulfing damaged neurons and cellular debris, reducing local inflammation, and thereby restoring neuronal function. Furthermore, *M2* microglia upregulate neurotrophic factors (e.g., BDNF), enhancing axonal regeneration and facilitating the repair and functional remodeling of injured neurons. Created in BioRender. Klyz, T. (2025) https://app.biorender.com/illustrations/69135c578eaccb8534812aeb?slideId=ad990900-8f7b-41e7-998e-06600f1ffad4.

Using MCAO rats, this study demonstrated ST909′s therapeutic efficacy during stroke recovery. As shown in Figure 12, ST909 significantly reduced infarct volume, attenuated cerebral edema, improved behavioral outcomes, suppressed neuroinflammation, and enhanced tissue repair-outperforming clinical standards (NBT/EDA). Mechanistically (Figure 11), ST909 upregulates the STING/IRF3 pathway, and promotes angiogenesis and oxidative resistance by releasing IFN-β, while phosphorylated IRF3 activates PI3K/Akt signaling. PI3K catalyzes PIP2-to-PIP3 con-version, enabling Akt phosphorylation and microglial polarization toward the *M2* phenotype, rebalancing *M1*/*M2* homeostasis.

*M2* microglia express TGF-β/Arg-1 while secreting IL-4/IL-10, enhancing phagocytosis to clear neural debris, mitigating inflammation, and protecting intact tissue [39,40]. Second, *M2*-type microglia rapidly infiltrate infarct and penumbral regions, removing cellular debris to reduce local inflammation and restore neuronal function [41,42]. Furthermore, *M2* microglia release BDNF/GDNF [25,26], guiding axonal regrowth along neurotrophic gradients to reconstruct neural circuits [43].

These findings align with established STING pathway activation patterns reported in previous stroke studies. Moderate STING/IRF3 pathway stimulation induces IFN-β production, promoting angiogenesis and neuroprotection [44,45]. Inacio et al. reported that endogenous IFN-β signaling attenuates local inflammation, regulates peripheral immune cells, and reduces infarct size and improved outcomes in a focal ischemia model [46]. Mathur et al. further demonstrated that IFN-β treatment activated IFNAR1 signaling, attenuating brain damage and restoring neurological function in MCAO rats [47]. Notably, Zhang et al. showed that cGAMP (a natural STING agonist) significantly decreased infarct volume and enhanced functional recovery in ischemic brain injury [27]. These findings establish STING activation as a promising preconditioning strategy for ischemic stroke, where IFN-related gene induction (particularly IFN-β) facilitates tissue repair, angiogenesis, and functional recovery. Li et al. revealed that cGAMP activates the STING/IRF3 and PI3K/AKT pathway, which subsequently stimulates angiogenesis and enhances neuronal survival during cerebral ischemia recovery [29]. The exploration of STING activation as a preconditioning strategy has also extended to synthetic STING agonists. It activates STING by stabilizing and accumulating cytosolic DNA, initiating a signaling cascade involving IRF3, and enhancing IFN associated gene expression [16]. STING pathway activation and subsequent induction of interferon-stimulated genes (ISGs), particularly IFN-β, play a pivotal role in enhancing tissue repair, stimulating neovascularization, and facilitating functional recovery after ischemic stroke.

Conversely, evidence shows that the cGAS/STING/IRF3 pathway exhibits dual roles in ischemic stroke, potentially mediating either neuroinflammatory apoptosis or functional recovery depending on activation context [48,49]. The cGAS/STING/IRF3 pathway mediates neuroinflammation during cerebral ischemia, where hyperactivation promotes excessive production of proinflammatory cytokines (e.g., TNF-α, IL-6) and chemokines, exacerbating cerebral damage [15,48]. Pharmacological inhibition of STING/IRF3 pathway attenuates neuroinflammation and confers therapeutic benefits in ischemic stroke models. This strategy offers multiple neuroprotective mechanisms [10,13,14]. However, STING/IRF3 pathway ablation may compromise CNS immune surveillance, increasing susceptibility to infections and impairing tissue repair capacity [50].

ST909 is a double-edged sword. At lower concentrations, it can repair damaged tissues in strokes; If the STING signaling pathway is excessively activated at high concentrations, it can trigger inflammation and cause brain tissue damage. This finding presents a significant challenge for clinical translation. To optimize the therapeutic window while balancing efficacy and safety, a mechanism-guided, stepwise dose exploration strategy should be implemented: First, preclinical data should be used to define potential therapeutic ranges, followed by early-phase clinical trials (e.g., Phase Ib) to meticulously assess pharmacodynamic markers (e.g., cytokine dynamics and brain injury biomarkers) and safety signals across low-to-mid dose ranges. Subsequently, Phase II trials should employ adaptive designs, incorporating real-time biomarker feedback to refine dosing, with a focus on identifying the “optimal biological dose” (OBD) rather than the maximum tolerated dose (MTD). This approach minimizes the risk of high-dose toxicity while preserving clinical benefit.

The middle cerebral artery occlusion (MCAO) model employed in this study is widely used in ischemic stroke research, but several limitations persist due to interspecies differences. First, the cerebrovascular anatomy of rodents differs significantly from that of humans. For instance, rodents exhibit a more complete Willis circle and enhanced collateral circulation capacity, whereas human patients often experience restricted collateral blood flow due to vascular variations or atherosclerosis [51]. These anatomical disparities may lead to discrepancies between the MCAO model and clinical observations regarding infarct volume, progression rate, and ischemic penumbra dynamics. Secondly, interspecies variations in immune responses may influence neuroinflammatory outcomes. Key differences include microglial activation thresholds, neutrophil infiltration patterns, and the release kinetics of pro-inflammatory cytokines (e.g., TNF-α and IL-1β) in rodents compared to humans [52]. Such divergence could contribute to the translational failure of neuroprotective strategies derived from animal studies. Furthermore, standard MCAO models typically use young, healthy animals, failing to replicate the impact of common comorbidities (e.g., hypertension, diabetes) on stroke outcomes in human patients [53]. While the MCAO model remains valuable for mechanistic investigations, these limitations may constrain the clinical generalizability of our findings. Future studies should employ non-human primate stroke models to better recapitulate human pathophysiology. Notably, ST909-edaravone combination therapy showed synergistic effects, providing new avenues for drug development. These findings establish a mechanistic framework for advancing ischemic stroke therapeutics.

## 4. Materials and Methods

### 4.1. Materials

ST909 (Cyclic Dinucleotide-Mg) was purchased by Hangzhou Orenstar Biomed Co., Ltd. (Hangzhou, China). Edaravone (EDA), (+)-borneol and Butylphthalide (NBT) were purchased from Sangon Biotech Co., Ltd. (Shanghai, China). 

### 4.2. Establishment and Grouping of Experimental Animal Models

Male SD rats (weighing 230–260 g, SPF grade) were purchased from Beijing Huafukang Biotechnology Co., Ltd., Beijing, China (license number: SCXK 2024-0003). One week prior to experiments, rats were acclimatized in standard housing facilities with ad libitum access to food and water under controlled environmental conditions (temperature: 22–25 °C; relative humidity: ~70%) on a 12 h light/dark cycle. All experimental procedures were approved by the Animal Ethics Committee of Fudan University and conducted in accordance with institutional guidelines for animal research. Following one week of acclimatization, rats with comparable body weights (within ±10 g) were randomly allocated into six groups (*n* = 6 per group). A rat model of focal cerebral ischemia–reperfusion was established according to the method described in reference [54]. Sedative rats received an intraperitoneal injection of 10% chloral hydrate. The common carotid artery (CCA), external carotid artery (ECA), and internal carotid artery (ICA) were bluntly dissected, and the ECA was ligated. A small incision was made at the ligation site, and a suture was advanced into the ICA through the opening. Upon encountering slight resistance, the suture was advanced until it reached the middle cerebral artery (MCA) and then secured in place. The MCAO thread is fabricated from moderately flexible monofilament nylon, which is precision-inserted through microscopic surgical manipulation. Its tip features a smooth polylysine-coated surface to ensure optimal performance during occlusion procedures. After 2 h of cerebral ischemia, the suture was carefully withdrawn to restore blood flow, thereby establishing a cerebral ischemia–reperfusion model. Postoperatively, drugs were administered immediately via the tail vein.

### 4.3. Dose-Dependent Effects of ST909 in Rats with Ischemic Brain Injury

The methods for model establishment, drug administration, and data analysis in the middle cerebral artery occlusion (MCAO) rat model followed the protocol described above. Twenty SD rats (weight: 250 ± 10 g) were randomly assigned to four groups. MCAO rats received a single tail vein injection of ST909 immediately after reperfusion. ST909 was administered at doses of 0.33 mg·kg^−1^ (low dose, L), 1.00 mg·kg^−1^ (medium dose, M), 3.00 mg·kg^−1^ (high dose 1, H1), and 9.00 mg·kg^−1^ (high dose 2, H2). MCAO rats were sedated under deep anesthesia 24 h after reperfusion, and brain tissues were collected for TTC staining to quantify the infarct volume. The optimal concentration of ST909 was determined by comparative analysis of ischemic lesion areas in rat brains across different administered doses.

### 4.4. The Therapeutic Effect of ST909 in Rats with Ischemic Brain Injury

The methods for middle cerebral artery occlusion (MCAO) model induction, drug administration, and data analysis followed the protocol detailed in Section 4. Rats were randomly assigned to sham-operated (G1), MCAO model (G2), reference drug 1 (NBT, G3), reference drug 2 (EDA, G4), ST909 (G5) and reference drug 3 (EDB, G6) groups. Sham-operated rats underwent exposure of the CCA, ICA, and ECA without filament insertion, other procedures matched the MCAO group. G1 received saline (3 mL·kg^−1^); G3: butylphthalide (20 mg·kg^−1^); G4: EDA (6 mg·kg^−1^); G5: ST909 (1 mg·kg^−1^); G6: EDA (6 mg·kg^−1^) + (+)-borneol (1.5 mg·kg^−1^). Drugs were administered via tail vein immediately post-reperfusion and once daily for 7 days. The MCAO model group received saline. Rat weight and behavioral scores were recorded daily; survival and recovery were monitored. Tissues were collected post-treatment for histopathological and statistical analysis. The therapeutic efficacy of ST909 was evaluated by comparative analysis of ischemic lesion volumes across treatment groups in a rat cerebral ischemia model, with first-line clinical drugs serving as positive controls.

### 4.5. TTC Staining-Based Measurement of Infarct Volume in Rat Brain

After inducing the MCAO model, rats were subjected to ischemia for 3, 7 days. Prior to sacrifice, anesthesia was administered. Blood was drained via the abdominal aorta to minimize residual blood interference with TTC staining. The whole brain was rapidly removed and frozen in −20 °C for 35 min and then sliced into 2 mm coronal sections, which were stained with 2% TTC at 37 °C in the dark for 30 min. Brain sections were then fixed in 4% paraformaldehyde for 24 h. The infarct area (white region) was quantified using a scanner and ImageJ 1.8.0 software, with the percentage calculated as: (Total white area/Total slice area) × 100% [55].

### 4.6. Assessment of Sensorimotor Functions in Rats with Ischemic Brain Injury

The balance beam test assesses motor function, muscle strength, and coordination in rats. As described in Reference [56], rats received one week of pre-surgical training until they could consistently traverse the beam (80 cm length, 2.5 cm width). Postoperatively, each rat underwent five consecutive daily trials. Neurological impairment was scored as follows (higher scores indicate greater impairment): 0 points: The subject passed through the balance beam without falling. 1 point: The subject passed through the balance beam but fell less than 50% of the time. 2 points: The subject passed through the balance beam but fell more than 50% of the time. 3 points: The subject passed through the balance beam, but the affected limb could not assist in movement. 4 points: The subject lay on the balance beam and was unable to move.

The screen test evaluates the forelimb grasping ability of rats. As described in Reference [57], post-surgical rats were placed at the center of the grid, and one end of the screen was gradually elevated until vertical (90°), positioned 45 cm above a foam pad. The duration of suspension was recorded (maximum: 5 s), with the time until landing defined as the suspension time. Scoring criteria were as follows: 0 points: The rat gripped the screen with its forepaws for 5 s without falling. 1 point: The rat briefly gripped the screen, slid partially, but did not fall. 2 points: The rat fell within 5 s. 3 points: The rat fell immediately upon screen rotation.

### 4.7. Multimodal MRI (T2WI/DWI) Reveals Spatiotemporal Changes in Rats with Ischemic Brain Injury

MRI scans were performed using a 7.0T magnetic resonance imaging system (7.0T-21, Shanghai Chenguang Medical Technology Co., Ltd., Shanghai, China) to observe changes in rats with brain injury before and after drug administration. Rats were anesthetized via isoflurane inhalation and positioned prone in the scanning coil. Coronal T2WI and DWI scans were performed separately. The T2WI scan parameters were as follows: TR = 3500 ms, TE = 52 ms, matrix size = 193 × 193, slice thickness = 1 mm, slice spacing = 0.1 mm, 12 slices, flip angle = 90°, FOV = 40 mm × 40 mm, NEX = 2. The DWI scanning parameters were as follows: TR = 3340 ms, TE = 110 ms, matrix size = 96 × 96; other parameters were identical to those of T2WI.

The apparent diffusion coefficient (ADC) was calculated as follows: First, the ranges of the normal area, penumbra, and lesion area were determined on T2WI. The ADC values of the corresponding regions on DWI were measured, and the ADC ratio was calculated to assess relative changes in brain functional recovery. FZ denotes the ADC value of the lesion area, NA denotes the ADC value of the normal area, and Pe denotes the ADC value of the penumbra. The normalized functional recovery (NFR) index reflects the recovery of the lesion area relative to the normal area, while the penumbra-to-normal ratio (PNR) reflects the recovery of the penumbra relative to the normal area.

$$NFR=\frac{FZ-NA}{NA}$$

$$PNR=\frac{Pe-NA}{NA}$$

### 4.8. Biochemical Detection of Inflammatory Factors, Neurotrophic Factors, and Cerebral Ischemia Markers in Rat Brain Tissue

Following MCAO induction, rats were sacrificed on days 3 and 7 post-procedure. Whole blood was collected from the abdominal aorta and left at room temperature for 30 min before centrifugation. The supernatant was collected and immediately tested for brain injury markers Hcy, S100β, and NSE. Whole brain tissue was collected from euthanized rats and mechanically homogenized to prepare a 10% homogenate. After centrifugation, the supernatant was collected for inflammatory factors TNF-α, IL-1β, IL-4, and IL-10, as well as neurotrophic factors BDNF, GDNF, and NGF-1.

### 4.9. Identification and Characterization of Microglial Subtypes in Rat Brain Tissue

Microglia, as key immune cells in the CNS, exhibit polarization states that significantly influence ischemic brain injury progression. We therefore employed qRT-PCR to quantify expression changes of microglial polarization markers following ST909 treatment.

GAPDH: Forward: 5′-CCAGCCCAGCAAGGATACTG-3′
Reverse: 5′-GGTATTCGAGAGAAGGGAGGGC-3′
MCP-1: Forward: 5’-ACGCTTCTGGGCCTGTTGTT-3’
Reverse: 5’-CCTGCTGCTGGTGATTCTCT-3’
CD32: Forward: 5’-TCTTCCTAAAGTATCCCCTGGA-3’
Reverse: 5’-AAAGGGAGCTCCTTAACATGC-3’
Arg-1: Forward: 5’-CCAGCCCAGCAAGGATACTG-3’
Reverse: 5’-GGTATTCGAGAGAAGGGAGGGC-3’
TGF-β: Forward: 5’-CACCTGCAAGACCATCGACA-3’
Reverse: 5’-CATAGTAGTCCGCTTCGGGC-3’

### 4.10. Immunofluorescence Analysis of STING/IRF3 and PI3K/AKT Pathway in Brain Tissue

The STING/IRF3 and PI3K/AKT pathways in the brain tissue of MCAO rats was examined by immunofluorescence. Rat brain tissue slices were deparaffinized and hydrated with xylene, antigen retrieval was performed and then blocked with 5% fetal bovine serum (FBS) albumin for 2 h. Primary rabbit antibodies (1:50, Beijing BioSense Biotechnology Co., Ltd., Beijing, China) were added and incubated overnight at 4 °C. The next day, after rewarming at 37 °C, the secondary antibody (1:100) was added dropwise, incubated at 37 °C for 2 h, and mounted with an anti-fade fluorescent mounting medium containing DAPI (protected from light). Images were captured using a fluorescence microscope (BX51, Olympus, Hachioji City, Japan), and the average fluorescence intensity was quantified using ImageJ software.

### 4.11. Data Analysis

Experimental data were plotted using GraphPad Prism 9.5 and expressed as mean ± standard deviation (mean ± SD). The ANOVA test was conducted using SPSS (version 19.0), followed by Tukey’s post hoc test with significance levels denoted as * *p* < 0.05 and ** *p* < 0.01. 

## 5. Conclusions

In this study, we employed the MCAO rat model to investigate the pharmacological effects and mechanisms of ST909 in treating ischemic brain injury. ST909 effectively reduces the infarct area, alleviates brain edema, and improves behavioral performance in rats with ischemic brain injury. The pharmacological mechanism underlying its anti-ischemic brain injury effects was elucidated. Under cerebral ischemia, ST909 activates the STING/IRF3 and IRF3/PI3K/Akt pathways to regulate microglial polarization. Specifically, it shifts microglia from the pro-inflammatory *M1* phenotype to the anti-inflammatory *M2* phenotype, thereby releasing anti-inflammatory factors to mitigate chronic inflammation. Additionally, ST909 enhances the secretion of neurotrophic factors, which repair damaged neurons, reduce ischemia-induced neuronal damage, and promote functional recovery of brain tissue.

The above pharmacodynamic results demonstrate that ST909 exhibits a superior therapeutic effect in the acute, subacute, and recovery phases compared to the clinical regimen of NBT, EDA or EDB. This study establishes ST909 as a promising therapeutic candidate for ischemic brain injury, with potential for clinical translation to improve stroke recovery outcomes. But its clinical translation faces several key challenges. Firstly, the pharmacokinetic (PK) of ST909 remain unclear, including potential competitive metabolism or altered blood–brain barrier penetration, which could significantly impact the safety profile and optimal dosing regimen of the combination therapy. Secondly, disease heterogeneity and variable drug responses in preclinical models (e.g., rodent MCAO models) may limit efficacy predictions, necessitating further validation in non-human primates or organoid models. Additionally, the optimal therapeutic concentration range and safe dosing window of ST909 require further precise definition through systematic experimental research.

Ultimately, overcoming translational barriers and maximizing the clinical benefits of this combination therapy will require an interdisciplinary approach integrating mechanistic research and adaptive trial design.

## Figures and Tables

**Table 1:** Statistical Analysis of rADC Values in Cerebral Ischemic Areas.

Group	0 Day		1 Day		3 Days		7 Days	
	NFR	PNR	NFR	PNR	NFR	PNR	NFR	PNR
MCAO	42.7 ± 3.7	22.5 ± 4.6	47.3 ± 5.1	24.4 ± 6.3	48.7 ± 2.1	26.1 ± 3.7	NA	NA
NBT	43.9 ± 4.6	23.1 ± 3.2	42.6 ± 3.6	23.3 ± 2.8	41.1 ± 3.8	24.1 ± 4.6	40.6 ± 3.9	23.9 ± 3.1
EDA	43.5 ± 2.7	24.4 ± 3.7	40.9 ± 4.7	23.6 ± 1.4	36.6 ± 5.1	22.9 ± 1.7	35.5 ± 4.2	21.1 ± 2.9
EDB	43.6 ± 1.9	23.1 ± 2.1	41.9 ± 5.2	21.3 ± 2.4	31.3 ± 5.2	15.2 ± 4.2	29.5 ± 4.7	12.9 ± 1.6
ST909	43.3 ± 5.1	24.4 ± 6.3	40.1 ± 3.2	22.9 ± 2.8	27.1 ± 3.1	14.3 ± 2.1	25.5 ± 3.7	8.96 ± 2.5

## Data Availability

The original data are available from the corresponding authors upon reasonable request.

## Supplementary Materials

The following supporting information can be downloaded at: https://www.mdpi.com/article/10.3390/ph18121775/s1, Figure S1: TTC staining analysis of cerebral infarction volume in ST909-treated MCAO rats across dose gradients; Figure S2: TTC staining analysis of cerebral infarction volume in different treatment group; Figure S3: Representative Brain MRI images; Table S1: Statistical comparison of cerebral infarction areas among treatment groups.

## Abbreviations

The following abbreviations are used in this manuscript:

MCAO	Middle cerebral artery occlusion e
MRI	Magnetic resonance imaging
NSE	Neuron-specific enolase
Hcy	Homocysteine
S100β	S100 calcium binding protein β
TGF-β	Transforming growth factor-β
MCP-1	Monocyte chemoattractant protein-1
EDA	Edaravone
EDB	Edaravone + (+)-borneol
NBT	3-n-Butylphthalide
