# Supplementary material for: Study on the Efficacy and Pharmacological Mechanism of Innate Immune STING Pathway Regulators in the Treatment of Ischemic Brain Injury
Source: Pharmaceuticals (Basel). 2025 Nov 21;18(12):1775. doi: 10.3390/ph18121775 (PMC12735853; doi:10.3390/ph18121775)
Supplement: Supplementary file 1 [file pharmaceuticals-18-01775-s001.zip › pharmaceuticals-3970637-supplementary.pdf]

pharmaceuticals

Article

Study on the Efficacy and Pharmacological Mechanism of Innate Immune STING Pathway Regulators in the Treatment of Ischemic Brain Injury

Chang Liu [1], Xiaoqing Wang [1], Yueru Zhang [2], Songli Yu [1] and Xiangshi Tan [1,*]

[1] Department of Chemistry, Fudan University, Shanghai 200433, China; 19110220106@fudan.edu.cn (C.L.); 20110220119@fudan.edu.cn (X.W.); 22110220101@fudan.edu.cn (S.Y.)
[2] Hangzhou Orenstar Biomed Co., Ltd., Hangzhou 314000, China; orenstar@163.com
* Correspondence: xstan@fudan.edu.cn; Tel.: +86-021-3124-9208

Academic Editors: Jasenka Mrsic-Pelcic, Anja Harej Hrkać and Stefania Chiappini

Received: 21 October 2025
Revised: 12 November 2025
Accepted: 18 November 2025
Published: 21 November 2025

Citation: Liu, C.; Wang, X.; Zhang, Y.; Yu, S.; Tan, X. Study on The Efficacy and Pharmacological Mechanism of Innate Immune STING Pathway Regulators in The Treatment of Ischemic Brain Injury. *Pharmaceuticals* **2025**, *18*, 1775. https://doi.org/10.3390/ph18121775

Copyright: © 2025 by the authors. Submitted for possible open access publication under the terms and conditions of the Creative Commons Attribution (CC BY) license (https://creativecommons.org/licenses/by/4.0/).

Figure S1–S3

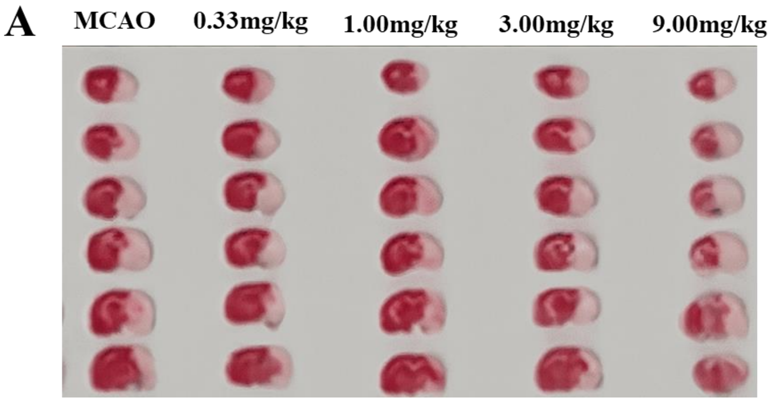

Figure S1. TTC staining analysis of cerebral infarction volume in ST909-treated MCAO rats across dose gradients: (A) Representative TTC-stained coronal brain sections from different treatment groups (doses: 0.33, 1.00, 3.00, 9.00 mg/kg). White areas indicate infarcted tissue.

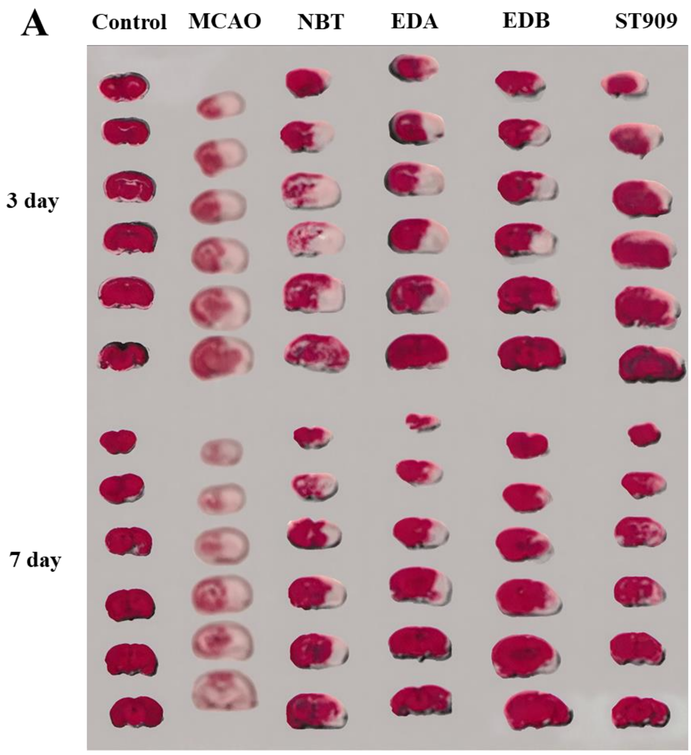

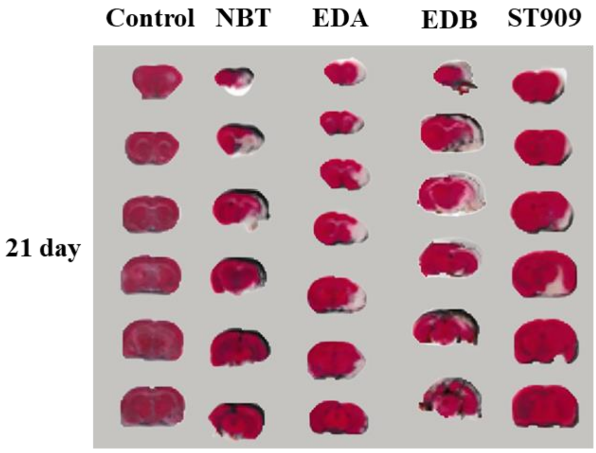

Figure S2. TTC staining analysis of cerebral infarction volume in different treatment group: (A) Representative TTC-stained coronal brain sections from different treatment groups in 3, 7, 21 days. White areas indicate infarcted tissue.

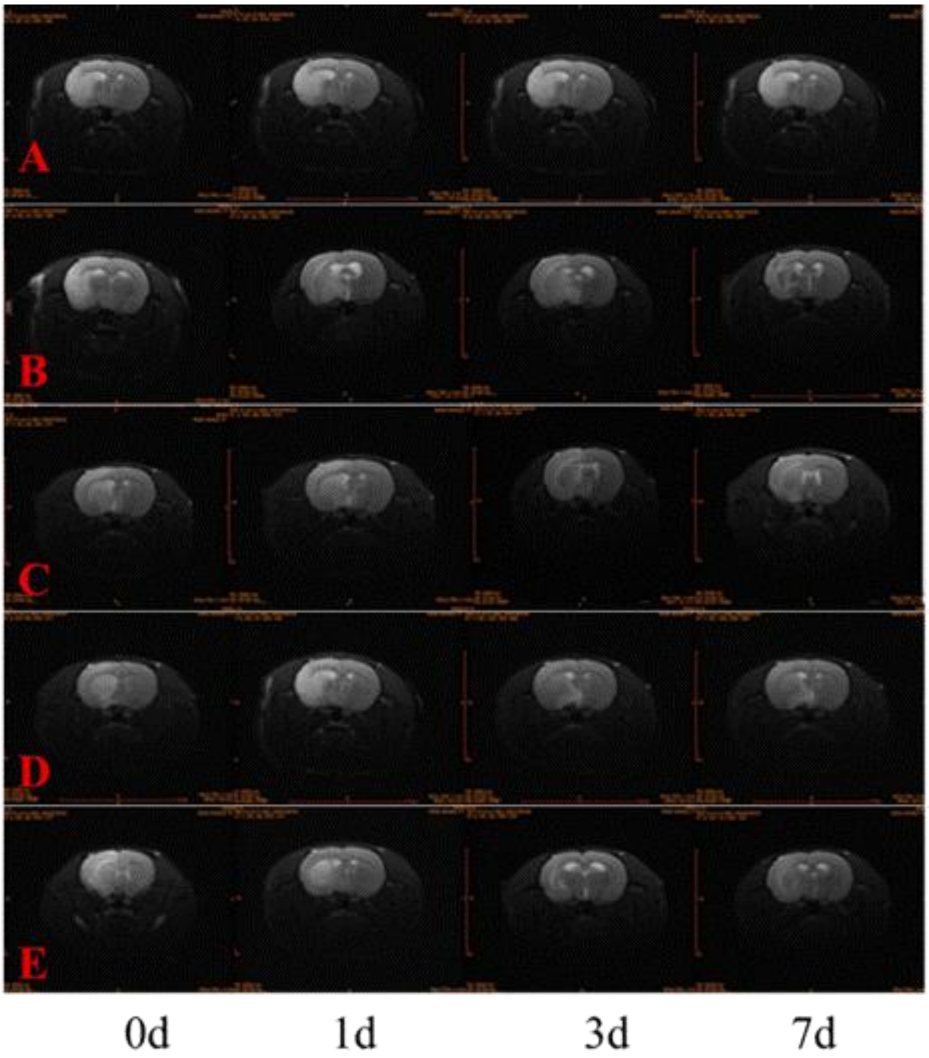

Figure S3. Representative Brain MRI images (A: MCAO, B: NBT, C: EDA, D: EDB, E: ST909).

Table S1

Table S1. Statistical comparison of cerebral infarction areas among treatment groups.

GROUP	3d		7d		21d	
	Area	Decreasing Percent	Area	Decreasing Ration	Area	Decreasing Ration
MCAO	47.3±1.9%		53.8±3.6%		NA	
NBT	35.2±5.7%	45.9%	29.5±7.2%	54.6%	15.2±6.2%	76.5%
EDA	27.2±4.3%	58.2%	23.3±5.7%	64.2%	12.1±3.8%	81.4%
ST909	21.7±5.1%	66.7%	13.1±4.6%	80.1%	4.6±3.2%	92.9%
EDB	26.9±7.4%	58.6%	19.2±6.8%	70.5%	12.1±5.4%	81.4%
